# Visual and High-Efficiency Secretion of SARS-CoV-2 Nanobodies with *Escherichia coli*

**DOI:** 10.3390/biom15010111

**Published:** 2025-01-12

**Authors:** Shuai Zhao, Wanting Zeng, Fang Yu, Pingping Xu, Chin-Yu Chen, Wanping Chen, Yanming Dong, Fei Wang, Lixin Ma

**Affiliations:** State Key Laboratory of Biocatalysis and Enzyme Engineering, Hubei Key Laboratory of Industrial Biotechnology, School of Life Sciences, Hubei University, Wuhan 430062, China; zhaoshuai@stu.hubu.edu.cn (S.Z.); 202011107010053@stu.hubu.edu.cn (W.Z.); yufang@stu.hubu.edu.cn (F.Y.); xupingping@stu.hubu.edu.cn (P.X.); chinyuchen@hubu.edu.cn (C.-Y.C.); wanpingchen@hubu.edu.cn (W.C.); wangfei@hubu.edu.cn (F.W.)

**Keywords:** nanobody, SARS-CoV-2, sfGFP, secretory expression, bispecific nanobody

## Abstract

Nanobodies have gained attention as potential therapeutic and diagnostic agents for severe acute respiratory syndrome coronavirus 2 (SARS-CoV-2) due to their ability to bind and neutralize the virus. However, rapid, scalable, and robust production of nanobodies for SARS-CoV-2 remains a crucial challenge. In this study, we developed a visual and high-efficiency biomanufacturing method for nanobodies with *Escherichia coli* by fusing the super-folder green fluorescent protein (sfGFP) to the N-terminus or C-terminus of the nanobody. Several receptor-binding domain (RBD)-specific nanobodies of the SARS-CoV-2 spike protein (S) were secreted onto the surface of *E. coli* cells and even into the culture medium, including Fu2, ANTE, mNb6, MR3-MR3, and n3113.1. The nanobodies secreted by *E. coli* retained equal activity as prior research, regardless of whether sfGFP was removed. Since some of the nanobodies bound to different regions of the RBD, we combined two nanobodies to improve the affinity. Fu2-sfGFP-ANTE was constructed to be bispecific for the RBD, and the bispecific nanobody exhibited significantly higher affinity than Fu2 (35.0-fold), ANTE (7.3-fold), and the combination of the two nanobodies (3.3-fold). Notably, Fu2-sfGFP-ANTE can be normally secreted into the culture medium and outer membrane. The novel nanobody production system enhances the efficiency of nanobody expression and streamlines the downstream purification process, enabling large-scale, cost-effective nanobody production. In addition, *E. coli* cells secreting the nanobodies on their surface facilitates screening and characterization of antigen-binding clones.

## 1. Introduction

The emergence of severe acute respiratory syndrome coronavirus 2 (SARS-CoV-2) in late 2019 caused profound global disruption, with devastating impacts on both public health and the economy [1,2,3]. The spike protein of SARS-CoV-2, particularly the receptor-binding domain (RBD), is essential for viral entry into host cells, as it binds to the host receptor angiotensin-converting enzyme 2 (ACE2) [4]. Consequently, the RBD has become a primary target for research aimed at blocking viral host cell interactions and mitigating infection [5,6], Several neutralizing monoclonal antibodies targeting the RBD have been developed, with promising results in preventing viral attachment and entry [7,8,9]. However, the development of scalable, cost-effective therapeutic alternatives remains a major challenge.

Nanobodies (VHHs), the heavy-chain-only antibodies found in Camelidae and sharks, offer compelling advantages over traditional antibodies as therapeutic agents [10]. These small, ~15 kDa proteins lack light chains and CH1 domains, making them highly versatile and capable of accessing epitopes that are often inaccessible to larger antibodies [11,12]. Additionally, nanobodies exhibit exceptional heat stability and can be expressed more efficiently than traditional antibodies [13]. Their rapid screening and high specificity make them ideal candidates for therapeutic applications, particularly in cases requiring fast-paced development [14,15,16]. In this study, we explored nanobodies with high affinity and neutralizing capabilities against the RBD of SARS-CoV-2. We identified several candidates, including MR3-MR3 [16], Fu2 [17], mNb6 [18], ANTE (PiN-21) [19,20], and n3113.1 [21].

*Escherichia coli* (*E. coli*) is a favored expression system for protein production, renowned for its well-characterized biology, ease of genetic modification, cost-effective cultivation, and rapid growth, enabling efficient and economical protein production [22,23]. However, producing nanobodies in the cytoplasm of *E. coli* poses significant challenges due to their reliance on conserved disulfide bonds. The reducing environment of the cytoplasm disrupts these bonds, destabilizing the secondary structure and often leading to insolubility caused by unfavorable interactions between folding intermediates [24]. Furthermore, while current expression systems are primarily aimed at enhancing protein or cytoplasm oxidation to promote disulfide bond formation, they frequently overlook streamlining downstream purification processes [25]. Therefore, it is crucial to develop a method that not only efficiently secretes and expresses these nanobodies but also simplifies the downstream purification process. This approach is essential for preserving their affinity against the RBD of SARS-CoV-2, ensuring their biological activity and therapeutic potential.

Protein secretory expression systems, which facilitate the display of proteins on the cell surface, are widely used for purification and functional analysis [26]. These systems commonly utilize signal peptides, such as OmpA or pelB, to translocate proteins across bacterial membranes [27,28]. However, in *E. coli*, a Gram-negative bacterium, the secretion of proteins is often impeded by the outer membrane, which serves as a significant barrier to efficient transport [29]. Moreover, these systems face challenges in ensuring proper protein folding and stability, particularly for proteins requiring complex disulfide bonds [30].

To overcome these limitations, super-folder green fluorescent protein (sfGFP) has emerged as a promising alternative. Its stable beta-barrel structure, intrinsic fluorescence, and fast folding enable the effective secretion of fused proteins through the outer membrane into the culture medium [31,32,33]. This method also supports the formation of functional disulfide bonds, essential for the activity of nanobodies. Additionally, sfGFP fusion tags improve protein solubility and stability in *E. coli*, making it a valuable tool for high-throughput protein expression [34]. In detail, we previously developed a cell-surface display system in recombinant *E. coli*, where PAP-Pb was fused to the N-terminus of sfGFP. This sfGFP-PAP-Pb fusion protein was secreted into the outer membrane, enhancing ATP synthesis through the use of intact cells [33]. Furthermore, Yan et al. demonstrated that sfGFP can facilitate the efficient expression and secretion of unspecific peroxygenases (UPOs), which are challenging to express in soluble and active forms in *E. coli* [35]. Lu et al. developed a secretion-based dual fluorescence assay (SDFA) for high-throughput screening of alcohol dehydrogenases (ADHs) [36]. SDFA is useful for determining specific activity and improving screening accuracy for ADHs, leading to a substantial improvement in their catalytic efficiency and facilitating the high-throughput screening process. These results demonstrate that sfGFP can function as a non-signal peptide to guide auto-secretion of nanobodies in *E. coli*. Bispecific nanobodies, capable of binding two different antigens simultaneously, offer new strategies for therapeutic intervention [37]. To enhance detection sensitivity, one approach involves using bispecific nanobodies with increased affinity [38]. These nanobodies can be engineered to target two separate tumor-associated antigens or receptors on the surface of tumor cells, enhancing immune responses and improving treatment efficacy [39]. Furthermore, bispecific nanobodies have also demonstrated robust neutralizing activities against SARS-CoV2 and high resistance to viral escape [40]. The incorporation of sfGFP not only boosts the production efficiency of these nanobodies but also simplifies monitoring and screening due to its fluorescent properties, which is crucial in high-throughput protein expression and selection. Leveraging sfGFP’s unique properties, we achieved efficient and straightforward expression, secretion, and preparation of functional nanobodies, demonstrating the potential of sfGFP in advancing biotechnology applications.

In this study, we introduce a highly effective system for nanobody secretion and expression, utilizing sfGFP as a fusion partner. By conjugating nanobodies with either the N- or C-terminus of sfGFP, we achieved real-time visualization of these nanobodies through green fluorescence, confirming their localization in both the outer membrane and the culture medium. Notably, the incorporation of sfGFP did not interfere with the binding activity of the nanobodies, thereby validating sfGFP as a secretion tag that augments protein yield without sacrificing functionality. Detailed structural analysis of the antigen–nanobody complex revealed that the Fu2 and ANTE nanobodies target distinct, non-overlapping epitopes on the RBD of SARS-CoV-2. Thus, we designed a bispecific nanobody, Fu2-sfGFP-ANTE, that could efficiently be secreted into both the culture medium and the outer membrane. This bispecific nanobody significantly enhanced synergistic binding potency for the SARS-CoV-2 S1 protein, outperforming the efficacy of the monomer nanobodies or the complex of the two monomer nanobodies, and demonstrated enhanced affinity and neutralizing potential. These findings underscore the potential of combining nanobodies with distinct epitopes to optimize neutralization efficacy.

## 2. Materials and Methods

### 2.1. Strains, Plasmids, and Reagents

*Escherichia coli* BL21(DE3) and DH5α were stored in the laboratory. pET-23a(+) and pET-23a(+)-sfGFP, used as the expression vectors, were maintained in our laboratory. Competent *E. coli* DH5α and *E. coli* BL21(DE3) were prepared in the laboratory. ELISA Stop solution, ELISA coating buffer (10×), bovine serum albumin (BSA), and TMB two-component substrate solution were purchased from Solarbio. HRP-conjugated mouse anti-HA-Tag mAb (AE025) was purchased from ABclonal (Wuhan, China). The primers used in the experiments (Table 1) were synthesized by Shanghai Sangon Biological Engineering Technology & Services Co., Ltd. (Shanghai, China).

### 2.2. Construction of Cloning Vector

**Figure 1:**
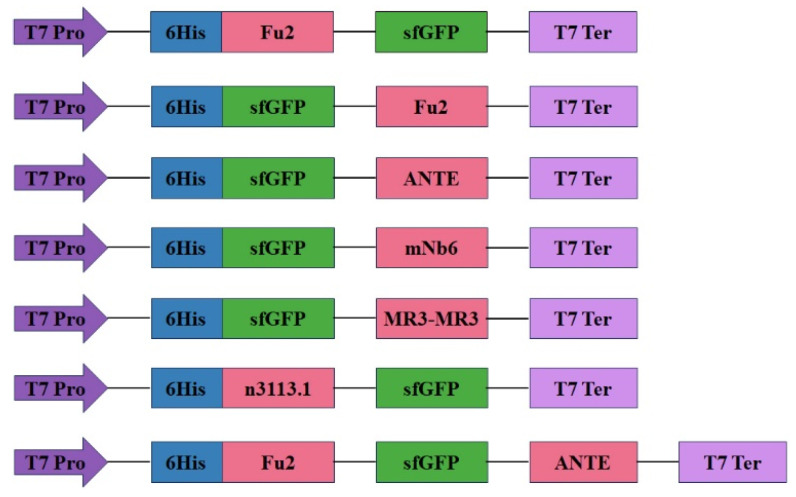
Construction of recombinant plasmids for the cell surface display of pET23a-Fu2-sfGFP, pET23a-sfGFP-Fu2, pET23a-sfGFP-ANTE, pET23a-sfGFP-mNb6, pET23a-sfGFP-MR3-MR3, pET23a- n3113.1-sfGFP, and pET23a-Fu2-sfGFP-ANTE. T7 Pro: T7 promoter; T7 Ter: T7 terminator.

The plasmid pET-23a was linearized by PCR using primers 23a-F and 23a-R (Table 1). The target genes Fu2, ANTE, mNb6, MR3-MR3, and n3113.1 were synthesized and cloned into the vector using primers (Table 1) with a 15 nt homology sequence as per previous research [41]. The recombinants were identified by sequencing. We named these plasmids pFu2-sfGFP, psfGFP-Fu2, psfGFP-ANTE, psfGFP-mNb6, psfGFP-MR3-MR3, pn3113.1-sfGFP, and pFu2-sfGFP-ANTE, as shown in Figure 1.

### 2.3. Protein Expression and Purification

The plasmids and strains used in this research are shown in Table 2. The recombinants E-Fu2-sfGFP, E-sfGFP-Fu2, E-sfGFP-ANTE, E-sfGFP-mNb6, E-sfGFP-MR3-MR3, E-n3113.1-sfGFP, and E-Fu2-sfGFP-ANTE were transformed into *E. coli* BL21(DE3) competent cells. Positive clones were selected and inoculated into 100 mL LB medium with 50 µg·mL^−1^ ampicillin, then cultured at 220 rpm and 37 °C. Upon reaching an optical density at 600 nm (OD600) of 0.6, protein expression was induced by adding 0.5 mM IPTG and transferring the culture to a shaker at 18 °C or 28 °C for various durations. Cells were harvested by centrifugation (15,000× *g*, 5 min, 4 °C), the medium was collected, and the pellet was completely resuspended in 10 mL of TEN washing buffer (50 mM Tris-HCl, 5 mM EDTA, and 50 mM NaCl pH 8.0) [29,33]. The mixture was incubated at 4 °C overnight. After centrifugation at 15,000× *g* for 10 min, the supernatant and medium were analyzed with sodium dodecyl sulfate–polyacrylamide gel electrophoresis (SDS-PAGE).

For the purification of nanobodies, we initially diluted the EDTA concentration to less than 1 mM using a buffer of 50 mM Tris-HCl and 50 mM NaCl at pH 8.0. This adjustment facilitated subsequent protein purification using Ni-NTA resin. After allowing batch binding for 1 h, the resin beads were washed with PBS containing a gradient of imidazole.

The buffer was then exchanged for PBS for Fu2-sfGFP, sfGFP-Fu2, sfGFP-ANTE, n3113.1-sfGFP, and Fu2-sfGFP-ANTE. For sfGFP-MR3-MR3, the buffer used was 20 mM Tris-HCl and 500 mM NaCl pH 8.0, while for sfGFP-mNb6, it was 20 mM HEPES and 150 mM NaCl pH 7.5.

To obtain nanobodies devoid of sfGFP, human rhinovirus 3C protease (HRV 3C) and small ubiquitin-related modifier (SUMO) protease were employed to cleave the sfGFP tag. The sfGFP or nanobody was captured using Ni-NTA resin, and nanobodies were collected in either the supernatant or the eluted buffer.

### 2.4. Western Blotting

The expression of proteins was assessed by Western blotting. Protein samples were separated with 12% or 15% SDS-PAGE gels and subsequently transferred onto polyvinylidene fluoride membranes. After electrophoresis, the membrane was blocked with 5% skim milk solution and then probed with His tag antibody (mouse monoclonal antibody, ABclonal) and horseradish peroxidase (HRP)-conjugated secondary antibody (mouse monoclonal antibody, ABclonal), each diluted 5000-fold in 5% skim milk.

Target proteins were visualized using a Western chemiluminescent HRP substrate (Millipore, Burlington, MA, USA) following standard protocols.

### 2.5. Enzyme-Linked Immunosorbent Assay (ELISA)

The SARS-CoV-2 coronavirus spike glycoprotein S1 (S1 protein) (Wuhan Huamei Biotech Co., Ltd., Wuhan, China) was diluted to a final concentration of 1 µg·mL^−1^, then coated onto 96-well plates and incubated at 4 °C overnight. Maltose-binding protein (MBP) was used as a negative control [42]. The samples were washed with PBST and blocked with blocking buffer (PBST contains 1% BSA) at room temperature for 1 h. Nanobodies were continuously diluted to different concentration gradients and incubated at room temperature for 1 h. The samples were washed again and then incubated with HRP-conjugated mouse anti-HA-Tag mAb (ABclonal) and TMB substrate. Optical density (OD) was measured using a spectrophotometer at 450 nm and 630 nm.

## 3. Results

### 3.1. Expression and Purification of the Recombinant Proteins

**Figure 2:**
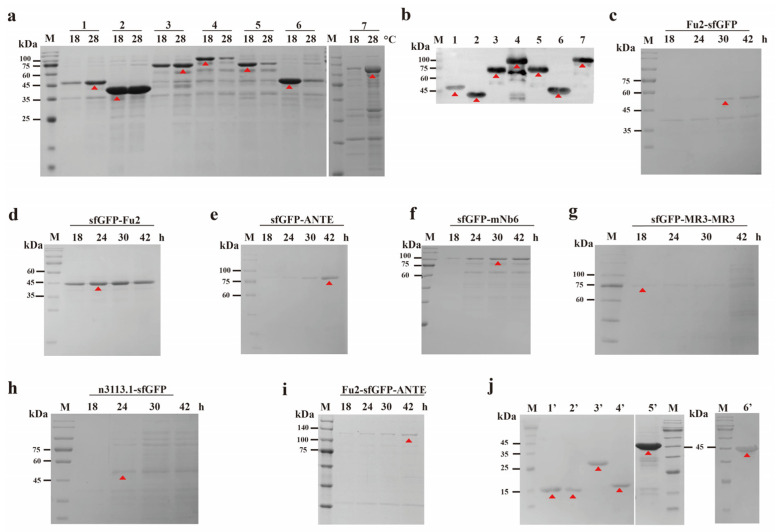
Analysis of nanobodies and sfGFP nanobody. (**a**) SDS-PAGE analysis of the outer membrane cell fraction. Lane 1: outer membrane fraction of Fu2-sfGFP after induction at 18 °C and 28 °C; lane 2: outer membrane fraction of sfGFP-Fu2 after induction at 18 °C and 28 °C; lane 3: outer membrane fraction of sfGFP-ANTE after induction at 18 °C and 28 °C; lane 4: outer membrane fraction of sfGFP-mNb6 after induction at 18 °C and 28 °C; lane 5: outer membrane fraction of sfGFP-MR3-MR3 after induction at 18 °C and 28 °C; lane 6: outer membrane fraction of n3113.1-sfGFP after induction at 18 °C and 28 °C; lane 7: outer membrane fraction of Fu2-sfGFP-ANTE after induction at 18 °C and 28 °C. (**b**) Western blotting for purified nanobodies. Lane 1, 2, 3, 4, 5, 6, and 7 correspond to Fu2-sfGFP, sfGFP-Fu2, sfGFP-ANTE, sfGFP-mNb6, sfGFP-MR3-MR3, n3113.1-sfGFP, and Fu2-sfGFP-ANTE. (**c**–**i**) SDS-PAGE analysis of the culture medium for the corresponding strains at different times (18, 24, 30, and 42 h). c, d, e, f, g, h and i correspond to E-Fu2-sfGFP, E-sfGFP-Fu2, E-sfGFP-ANTE, E-sfGFP-mNb6, E-sfGFP-MR3-MR3, E-n3113.1-sfGFP, and E-Fu2-sfGFP-ANTE. respectively. (**j**) SDS-PAGE analysis of the nanobodies without sfGFP. Lane 1’: Fu2: HRV 3C protease was used to remove the sfGFP tag from Fu2-sfGFP; lane 2’: Fu2: HRV 3C protease was used to remove the sfGFP tag from sfGFP-Fu2; lane 3’: MR3-MR3: SUMO protease was used to remove the sfGFP tag from sfGFP-MR3-MR3; lane 4’: n3113.1: HRV 3C protease was used to remove the sfGFP tag from n3113.1-sfGFP; lane 5’: ANTE: HRV 3C protease was used to remove the sfGFP tag from sfGFP-ANTE; lane 6’: mNb6: SUMO protease was used to remove the sfGFP tag from sfGFP-mNb6. M: Marker.

According to the literature, nanobodies such as MR3-MR3, Fu2, mNb6, ANTE, and n3113.1 demonstrate high affinity and neutralizing capabilities against the RBD [16,17,18,19,20]. This suggests that these nanobodies hold significant potential for future therapeutic and detection applications. Consequently, we constructed a series of plasmids and developed several recombinant strains, including E-Fu2-sfGFP, E-sfGFP-Fu2, E-sfGFP-ANTE, E-sfGFP-mNb6, E-sfGFP-MR3-MR3, E-n3113.1-sfGFP, and E-Fu2-sfGFP-ANTE. These strains were cultured as described in the Methods section. Proteins were extracted and analyzed by SDS-PAGE. As shown in Figure 2a, sfGFP nanobodies appeared in the fraction of the outer cell membrane. Fu2-sfGFP, sfGFP-ANTE, and Fu2-sfGFP-ANTE were mainly observed after induction at 28 °C, while sfGFP-mNb6, sfGFP-MR3-MR3, and n3113.1-sfGFP were mainly observed after induction at 18 °C. sfGFP-Fu2 was observed after induction at both 18 °C and 28 °C. SDS-PAGE analysis showed that relevant protein bands appeared near the predicted molecular weight (Figure 2a), which was further confirmed by Western blot analysis with purified nanobodies (Figure 2b).

In *E. coli*, VHHs expressed in the periplasm typically yield dozens of milligrams per liter, whereas cytoplasmic expression in strains such as SHuffle and BL21(DE3) can achieve up to 200 mg·L^−1^. Yeast systems, particularly *S. cerevisiae*, have demonstrated the potential to reach titers of approximately 600 mg·L^−1^ through fed-batch fermentation. Nonetheless, the yeast system encounters challenges, including the risk of unwanted N-glycosylation, which can impact VHH antigen binding and increase immunogenicity [22]. The yield of the recombinant nanobodies extracted from the outer membrane was measured (Table 3), and the yield of the sfGFP-fusion nanobodies ranged from 188 to 335 mg·L^−1^. This result indicated that the nanobodies could be secreted into the cell membrane highly efficiently by fusing with sfGFP, presenting a significant advantage compared to MR3 expressed in *E. coli* MC1061 cells with 18.8 mg·L^−1^ [16].

Notably, most of the nanobodies were secreted into the culture medium, as shown in Figure 2c–i. sfGFP-Fu2 was mainly observed after induction at 18 °C, while the others were mainly observed after induction at 28 °C. To separate the sfGFP from the nanobodies, HRV 3C or SUMO protease was used to remove the sfGFP tag, and the purified nanobodies are displayed in Figure 2j. According to previous research, some of the nanobodies and sfGFP are thermostable [43,44], and thus we tried to purify the sfGFP-fusion nanobodies with heat treatment. Most of the impurity proteins were able to be removed after heat treatment at 80 °C for 30 min, and the fluorescence intensity of the recombinant proteins was comparable after the heat treatment (Supplementary Materials Figure S1).

### 3.2. Binding Capacity to S1 of the Recombinant Nanobodies

**Figure 3:**
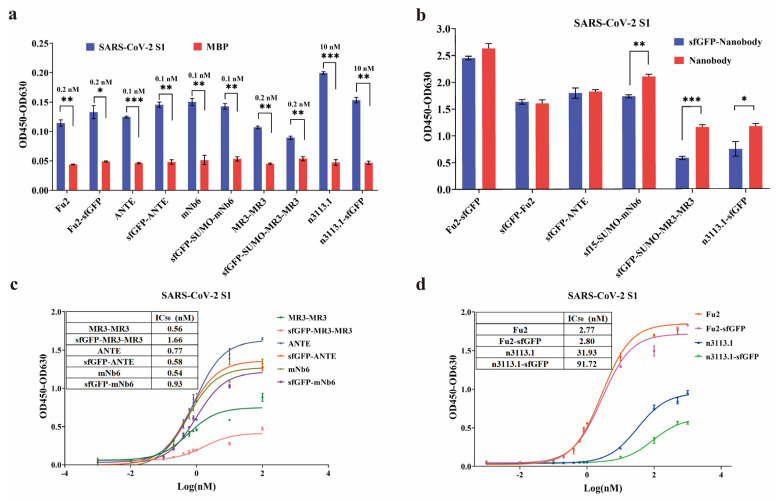
Indirect ELISA to identify the affinity of nanobodies to SARS-CoV-2 S1-RBD. (**a**) Binding affinities of various nanobodies and sfGFP-fusion nanobodies against the S1 protein or the negative control MBP at 0.1 nM, 0.2 nM, or 10 nM. (**b**) Binding affinities of various nanobodies and sfGFP-fusion nanobodies against the S1 protein at 1 uM. (**c**,**d**) Binding affinities of various nanobodies and sfGFP-fusion nanobodies against the S1 protein at various concentrations. Results shown as means ± SD of three parallel replicates (* *p* < 0.05, ** *p* < 0.01, *** *p* < 0.001).

The affinity of the nanobodies was evaluated using indirect ELISA. All of the recombinant nanobodies generated high absorbance values at 450 nm (Figure 3a), and the IC_50_ of nanobodies was lower than 3 nM, except the n3113.1, demonstrating that the recombinant nanobodies had high affinity against SARS-CoV-2 S1 (Figure 3c,d). Additionally, we compared the affinity of the nanobodies with and without the sfGFP tag. For Fu2, mNb6, and ANTE, the sfGFP fusion nanobodies showed comparable affinity with the monomer nanobodies, while MR3-MR3 and n3113.1 showed higher affinity when sfGFP was removed from the recombinant nanobodies (Figure 3b–d).

### 3.3. Multi-Epitope Nanobody with Enhanced Affinity

**Figure 4:**
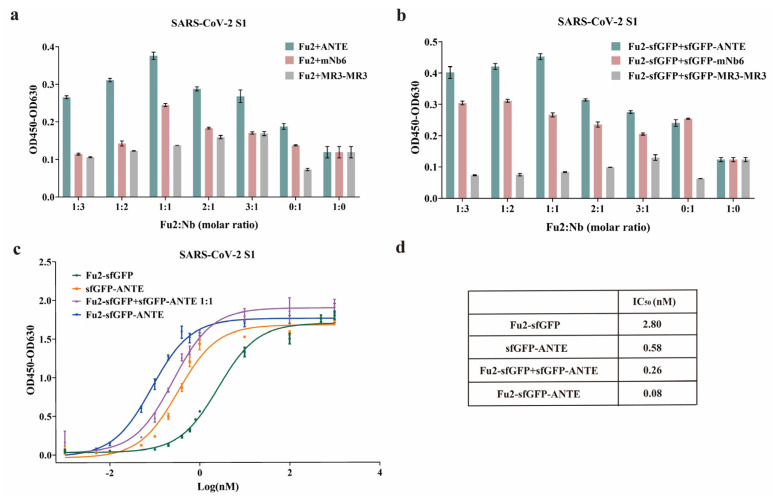
Two different nanobodies with SARS-CoV-2. (**a**) Binding affinities of nanobody mixture without sfGFP at 0.2 nM. Statistical analysis was performed using a *t*-test. Significant differences are indicated as *p* < 0.05 or *p* < 0.01. Specifically, Fu2:ANTE at molar ratios of 1:3 and 3:1 showed a significant difference (*p* < 0.05) compared to the 0:1 and 1:0 molar ratios, and at molar ratios of 1:2, 1:1, and 2:1, showed a highly significant difference (*p* < 0.01) compared to the 0:1 and 1:0 molar ratios. Fu2:mNb6 at molar ratios of 1:1 and 2:1 showed a highly significant difference (*p* < 0.01) compared to the 0:1 ratio, and at a 1:1 molar ratio, also showed a significant difference (*p* < 0.05) compared to the 1:0 molar ratio. Fu2: MR3-MR3 at molar ratios of 1:3, 1:2, 1:1, 2:1, and 3:1 showed a highly significant difference (*p* < 0.01) compared to the 0:1 molar ratio. (**b**) Binding affinities of nanobody mixture with sfGFP at 0.4 nM, specifically, Fu2-sfGFP:sfGFP-ANTE at molar ratios of 1:3 and 2:1, showed a significant difference (*p* < 0.05) compared to the 0:1 molar ratio and a highly significant difference (*p* < 0.01) compared to the 1:0 molar ratio. At molar ratios of 1:2 and 1:1 it showed a highly significant difference (*p* < 0.01) compared to the 0:1 and 1:0 molar ratios, and at a 3:1 molar ratio, showed a highly significant difference (*p* < 0.01) compared to the 1:0 molar ratio. Fu2-sfGFP:sfGFP-mNb6 at a 1:3 molar ratio showed a significant difference (*p* < 0.05) compared to the 0:1 molar ratio and a highly significant difference (*p* < 0.01) compared to the 1:0 molar ratio, at a 1:2 molar ratio, showed a highly significant difference (*p* < 0.01) compared to the 0:1 and 1:0 molar ratios, and at molar ratios of 1:1 and 2:1, showed a highly significant difference (*p* < 0.01) compared to the 1:0 molar ratio. Fu2-sfGFP:sfGFP-MR3-MR3 at molar ratios of 1:3, 1:2, 1:1, 2:1, and 3:1 showed a significant difference (*p* < 0.05) compared to the 0:1 molar ratio. (**c**,**d**) Binding affinities of Fu2-sfGFP, sfGFP-ANTE, mixture, and Fu2-sfGFP-ANTE toward the S1 protein at various concentrations, as determined by ELISA. Results are shown as means ± SD of three parallel replicates.

It is possible to improve sensitivity in immunoassays utilizing multivalent antibodies with enhanced affinity [38]. A comparison analysis of the binding sites of nanobodies, conducted using AlphaFold3, revealed that the interaction interface of Fu2 and other nanobodies (ANTE, mNb6, and MR3-MR3) is significantly different (Supplementary Materials Figure S2) [45]. To evaluate the potential of multivalency in enhancing the affinity against the RBD, Fu2 was combined with other nanobodies at different molar ratios of 1:3, 1:2, 1:1, 2:1, and 3:1 in PBS. For nanobodies without sfGFP, the total protein content was 0.2 nM, while for the sfGFP-fusion nanobodies, the total protein content was 0.4 nM. The results indicated that the combination of nanobodies with different binding sites gained improved affinity against the RBD (Supplementary Materials Figures S3 and S4). Specifically, the Fu2 and ANTE nanobody complexes demonstrated superior affinity against the RBD (Figure 4a,b). Thus, Fu2 and ANTE were selected for further improvements.

Previously, it was reported that the binding affinity of the monomer nanobody can be improved by tandem linking [44,46,47]. To further improve the affinity, we positioned these two nanobodies at the two ends of sfGFP. The results demonstrated that the bispecific nanobody Fu2-sfGFP-ANTE was successfully secreted into both the culture medium and outer membrane (Figure 2a,i), highlighting this innovative approach to the design and preparation of bispecific nanobodies. ELISA analysis further revealed that Fu2-sfGFP-ANTE exhibited superior affinity compared to the monomer nanobodies or the complex of the two monomer nanobodies. (Figure 4c,d). Briefly, the affinity of Fu2-sfGFP-ANTE enhanced about 35.0, 7.3, and 3.3 times compared to Fu2-sfGFP, sfGFP-ANTE, and the complex of the two nanobodies, respectively.

## 4. Discussion

The ongoing coronavirus pandemic has highlighted the urgent need for scalable, effective vaccines, antibodies, and antiviral treatments [48,49,50]. However, the challenge of large-scale manufacturing of these therapeutics is substantial, particularly when endeavors are made to engineer them for multi-specificity, which can lead to significant cost increases [42]. In contrast, nanobodies offer a compelling attractive alternative to monoclonal antibodies for viral neutralization. The straightforward production, exceptional thermal stability, robust biochemical properties, and ease of functionalization and multimerization make nanobodies highly promising candidates for therapeutic applications [51,52,53,54].

In this research, sfGFP was designed as a secretion tag for efficient secretory expression of nanobodies. The fusion of nanobodies with sfGFP, either at the N-terminal, C-terminal, or even at both termini, enabled visibly secreted expression of the nanobodies. The fluorescence of sfGFP provides a robust and visual marker for real-time monitoring of the protein expression and purification processes. Additionally, the nanobodies were able to be secreted into the culture, which eliminates the necessity for cell lysis and minimizes hands-on time. The thermostability of sfGFP and nanobodies enables the high-efficiency removal of most impurity proteins with heat treatment, followed by one-step Ni-NTA purification, with which we achieved relatively highly pure and high-affinity nanobodies, significantly streamlining downstream purification procedures. Interestingly, the incorporation of sfGFP did not compromise the affinity of most nanobodies, suggesting its suitability as a visible and highly efficient strategy to produce high-affinity nanobodies.

Utilizing the secretory expression system, five nanobodies against the SARS-CoV-2 RBD domain were prepared, and all the nanobodies demonstrated similar affinity compared to previous reports. By adopting a multi-epitope strategy, we found that the combination of nanobodies with distinct binding sites significantly increased affinity against the SARS-CoV-2 S1 protein. Among these nanobodies, the combination of Fu2 and ANTE gained relatively high affinity. Considering the fusion of sfGFP did not reduce the affinity of Fu2 and ANTE, we designed a bispecific nanobody by linking Fu2 and ANTE with sfGFP. The Fu2-sfGFP-ANTE “sandwich” was secreted into both the outer membrane and the culture medium and the affinity of Fu2-sfGFP-ANTE reached 0.08 nM, which was 35.0-fold and 7.3-fold higher compared to Fu2-sfGFP and sfGFP-ANTE, respectively. This result highlights the potential of the secretory expression system for manufacturing high-affinity nanobodies for use in commercial diagnostics, therapeutic agents, and numerous research applications.

In this work, we developed an efficient nanobody biomanufacturing platform based on an sfGFP-driven secretory expression system. This approach resulted in more robust protein expression and purification, enabling high-throughput screening and efficient, scalable nanobody production. Combined with artificial intelligence (AI)-driven antibody design technology, the system will expedite VHH development.

However, our research mainly focused on the S1 subunit rather than the intact spike, highlighting a clear need for future studies to incorporate the full spike protein. Moreover, we are committed to continuing our in vivo assessments of nanobodies’ ability to neutralize viruses, especially those engineered with the sfGFP. These assessments could provide compelling evidence of their utility in biological research, thereby broadening their potential applications.

## 5. Conclusions

In summary, we developed a visual and high-efficiency nanobody secretory expression system that allows for nanobody production in an easy and cost-effective way. This system allows the secretion of any target nanobody into the cell membrane or the culture medium, resulting in high-yield production of these nanobodies in a bioactive and well-folded form. In conclusion, the results of this study position the sfGFP-driven secretory expression as a key enabling technology for future research and development of nanobodies, and with further advances, potentially for other interesting proteins with disulfide bonds.

## Figures and Tables

**Table 1:** Primers used in this study.

Gene	Primer	Sequence (5′-3′)
Fu2-sfGFP	Fu2-NF	CACCATCATCATCATCATCAGGTTCAGCTGGTTGAAAGC
	Fu2-NR	AGCAGCCGGATCTCATTTATACAGTTCATCCATGCCC
sfGFP-Fu2	Fu2-CF	CACCATCATCATCATCATATGGTGAG
	Fu2-CR	AAGCGTAATCCGGAACATCATACGGGTAGCTGCTAACGGTAACCTGG
sfGFP-ANTE	ANTE-F	CACCATCATCATCATCATATGGTGAG
	ANTE-R	AAGCGTAATCCGGAACATCATACGGGTAAGAGCTAACGGTCACTTGC
sfGFP-mNb6	mNb6-F	CACCATCATCATCATCATATGGTGAG
	mNb6-R	GCTGCCGCCGCCGCCGCTACTAACTGTAACTTGTGTTCCC
sfGFP-MR3-MR3	MR3-F	CACCATCATCATCATCATATGGTGAG
	MR3-R	AGCAGCCGGATCTCACTATGCATAATCCGGAACATCATACG
n3113.1-sfGFP	n3113-F	CACCATCATCATCATCATGAGGTTCAACTAGTAGAATCAGGTGG
	n3113-R	AGCAGCCGGATCTCATTTATACAGTTCATCCATGCCC
pET-23a	23a-F	TGAGATCCGGCTGCTAACAA
	23a-R	ATGATGATGATGATGGTGCATATGTATAT

**Table 2:** Plasmids and strains used in this study.

Plasmid/Strain	Description
pET-23a	Vector for expression proteins, T7 promoter, Amp^r^
pFu2-sfGFP	pET-23a encoding *Fu2-sfGFP*, Amp^r^
psfGFP-Fu2	pET-23a encoding *sfGFP-Fu2*, Amp^r^
psfGFP-ANTE	pET-23a encoding *sfGFP-ANTE*, Amp^r^
psfGFP-mNb6	pET-23a encoding *sfGFP-mNb6*, Amp^r^
psfGFP-MR3-MR3	pET-23a encoding *sfGFP-MR3-MR3*, Amp^r^
pn3113.1-sfGFP	pET-23a encoding *n3113.1-sfGFP*, Amp^r^
pFu2-sfGFP-ANTE	pET-23a encoding *Fu2-sfGFP-ANTE*, Amp^r^
Strain	
E-Fu2-sfGFP	*E. coli* BL21(DE3) (pFu2-sfGFP)
E-sfGFP-Fu2	*E. coli* BL21(DE3) (psfGFP-Fu2)
E-sfGFP-ANTE	*E. coli* BL21(DE3) (psfGFP-ANTE)
E-sfGFP-mNb6	*E. coli* BL21(DE3) (psfGFP-mNb6)
E-sfGFP-MR3-MR3	*E. coli* BL21(DE3) (psfGFP-MR3-MR3)
E-n3113.1-sfGFP	*E. coli* BL21(DE3) (pn3113.1-sfGFP)
E-Fu2-sfGFP-ANTE	*E. coli* BL21(DE3) (pFu2-sfGFP-ANTE)

All plasmids are reported for the first time in this study. The “r” in Amp^r^ stands for “resistance”, indicating Ampicillin resistance.

**Table 3:** The expression yields (mg/L) of the nanobodies.

Nanobodies	Number of Cysteines	MW (Target Protein) (kDa)	Secretion Production (mg/L)
Fu2-sfGFP	4	45.1	188
sfGFP-Fu2	4	44.1	307
sfGFP-ANTE	8	70.9	335
sfGFP-mNb6	8	82.2	331
sfGFP-MR3-MR3	6	69.9	294
n3113.1-sfGFP	4	42.7	235
Fu2-sfGFP-ANTE	10	97.9	272

MR3-MR3 is bivalent, ANTE and mNb6 are trivalent.

## Data Availability

All relevant data of this study are presented. Additional data will be provided upon request.

## Supplementary Materials

The following supporting information can be downloaded at https://www.mdpi.com/article/10.3390/biom15010111/s1. Figure S1: Analysis of sfGFP-fused nanobodies pre- and post-heat treatment; Figure S2: The complex structure of RBD and nanobodies; Figure S3: Binding affinities of nanobody mixture without sfGFP; Figure S4: Binding affinities of nanobody mixture with sfGFP.
