# Supplementary material for: Visual and High-Efficiency Secretion of SARS-CoV-2 Nanobodies with Escherichia coli
Source: Biomolecules. 2025 Jan 12;15(1):111. doi: 10.3390/biom15010111 (PMC11762740; doi:10.3390/biom15010111)
Supplement: Supplementary file 1 [file biomolecules-15-00111-s001.zip › biomolecules-3385463-supplementary.pdf]

Supplementary Figures:

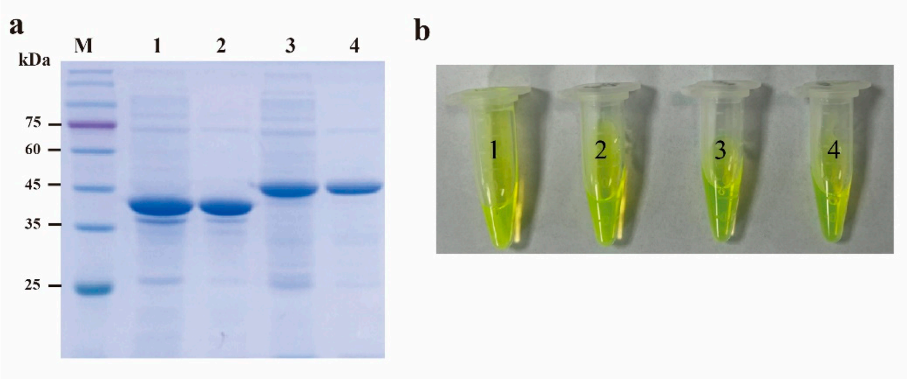

Figure S1. Analysis of sfGFP-fused nanobodies pre- and post-heat treatment. (**a**) SDS-PAGE analysis was conducted on the outer membrane cell fractions of sfGFP-Fu2 and n3113.1-sfGFP, comparing the pre- and post-heat treatment samples for each protein. (**b**) The green fluorescence of the outer membrane cell fractions from sfGFP-Fu2 and n3113.1-sfGFP was compared between pre- and post-heat treatment samples. 1: Outer membrane fraction of sfGFP-Fu2; 2: Outer membrane fraction of sfGFP-Fu2 after heat treatment at 80 °C for 30 min; 3: Outer membrane fraction of n3113.1-sfGFP; 4: Outer membrane fraction of n3113.1-sfGFP after heat treatment at 80 °C for 30 min. M: Marker.

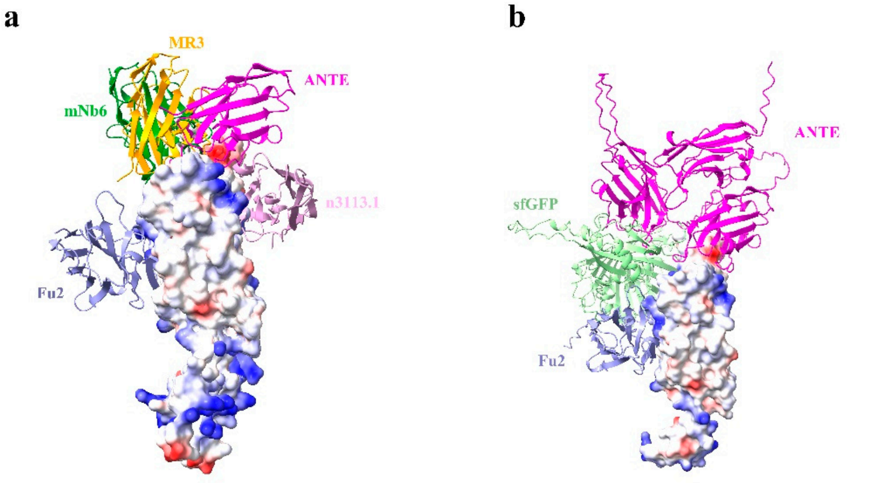

Figure S2. The complex structure of RBD and nanobodies. (**a**) The complex structure of five nanobodies and RBD predicted by AlphaFold3. (**b**) The complex structure of Fu2-sfGFP-ANTE and RBD predicted by AlphaFold3.

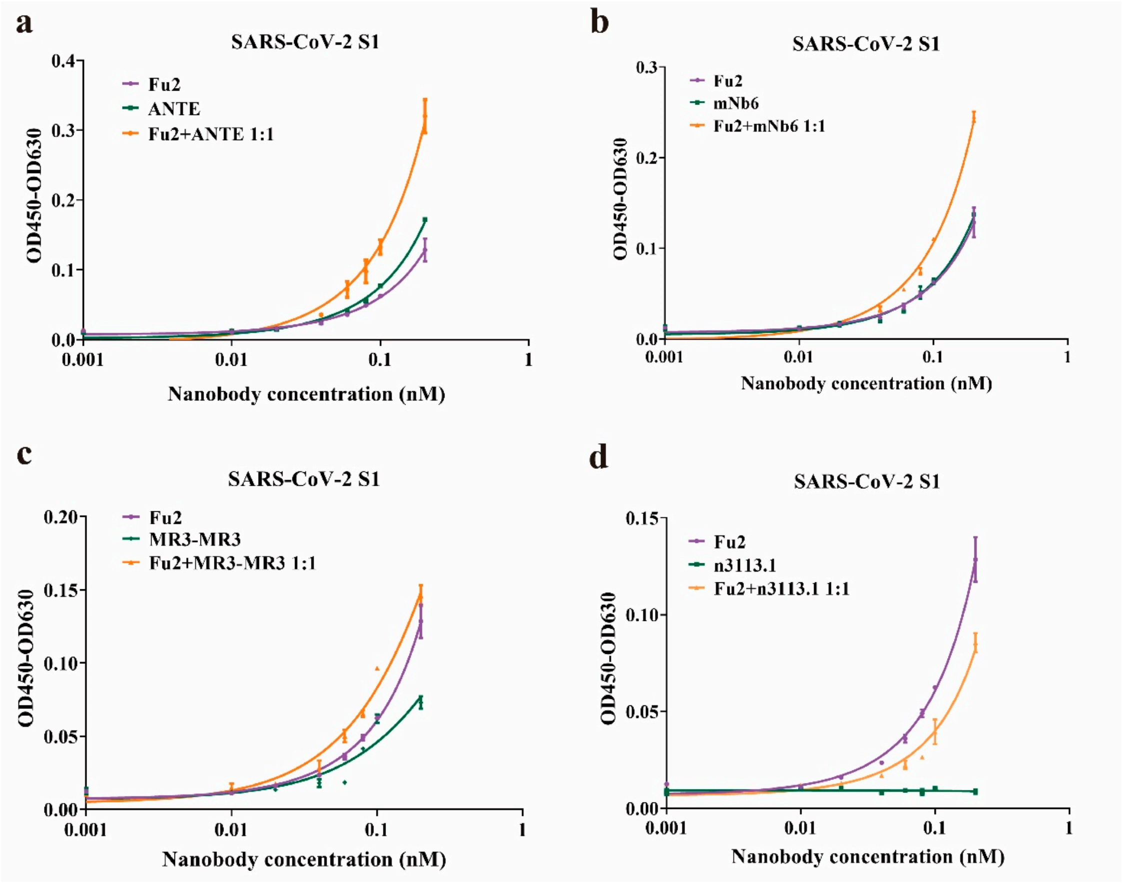

Figure S3. Binding affinities of nanobody mixture without sfGFP. (**a**) Binding affinities of Fu2, ANTE and nanobody mixture toward the S1 protein at various concentrations. (**b**) Binding affinities of Fu2, mNb6 and nanobody mixture toward the S1 protein at various concentrations. (**c**) Binding affinities of Fu2, MR3-MR3 and nanobody mixture toward the S1 protein at various concentrations. (**d**) Binding affinities of Fu2, n3113.1 and nanobody mixture toward the S1 protein at various concentrations.

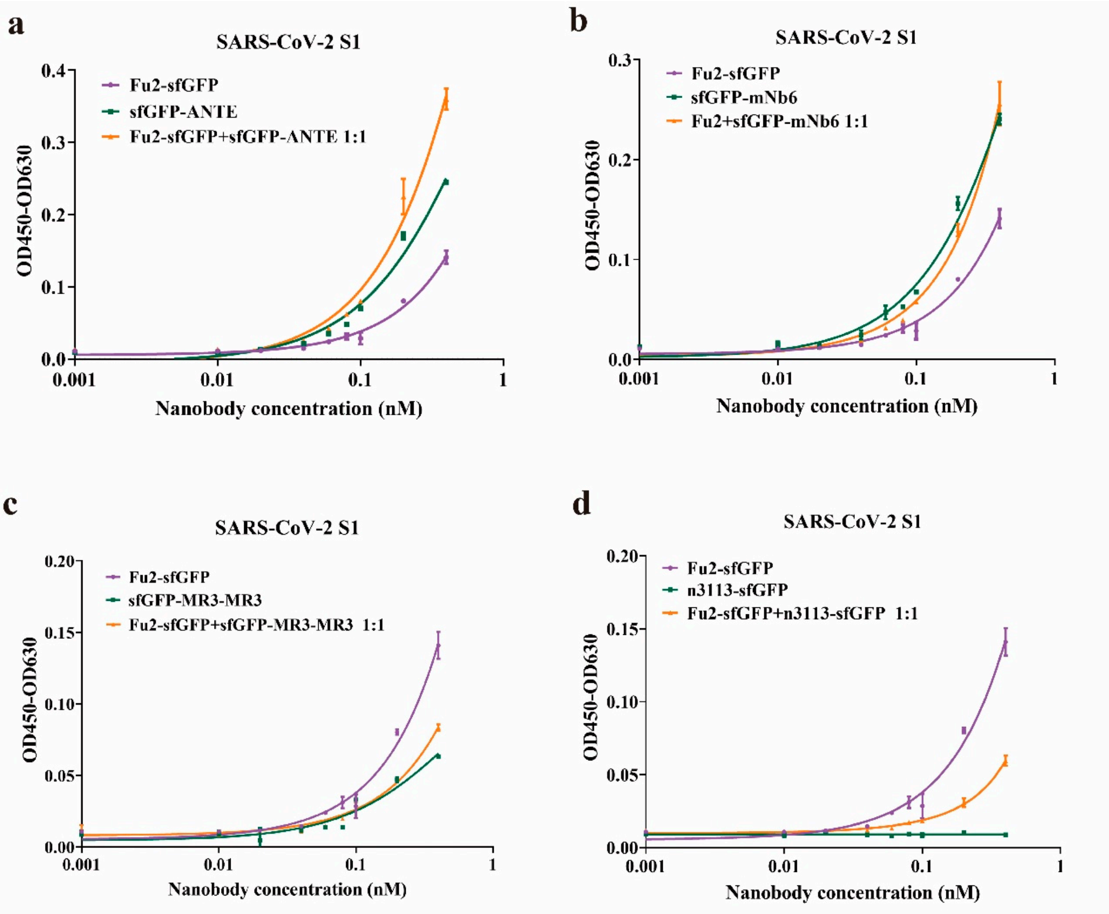

Figure S4. Binding affinities of nanobody mixture with sfGFP. (**a**) Binding affinities of Fu2-sfGFP, sfGFP-ANTE and nanobody mixture toward the S1 protein at various concentrations. (**b**) Binding affinities of Fu2-sfGFP, sfGFP-mNb6 and nanobody mixture toward the S1 protein at various concentrations. (**c**) Binding affinities of Fu2-sfGFP, sfGFP-MR3-MR3 and nanobody mixture toward the S1 protein at various concentrations. (**d**) Binding affinities of Fu2-sfGFP, n3113.1-sfGFP and nanobody mixture toward the S1 protein at various concentrations.
